# Cough Aerosol Cultures of *Mycobacterium tuberculosis*: Insights on TST / IGRA Discordance and Transmission Dynamics

**DOI:** 10.1371/journal.pone.0138358

**Published:** 2015-09-22

**Authors:** Edward C. Jones-López, Laura F. White, Bruce Kirenga, Francis Mumbowa, Martin Ssebidandi, Stephanie Moine, Olive Mbabazi, Gerald Mboowa, Irene Ayakaka, Soyeon Kim, Christina S. Thornton, Alphonse Okwera, Moses Joloba, Kevin P. Fennelly

**Affiliations:** 1 Section of Infectious Diseases, Department of Medicine, Boston Medical Center and Boston University School of Medicine, Boston, Massachusetts, United States of America; 2 Department of Biostatistics, Boston University School of Public Health, Boston, Massachusetts, United States of America; 3 Department of Medicine, Makerere University College of Health Sciences, Kampala, Uganda; 4 Department of Microbiology, Makerere University College of Health Sciences, Kampala, Uganda; 5 Makerere University–Boston Medical Center Research Collaboration, Kampala, Uganda; 6 Infectious Diseases Institute, Makerere University College of Health Sciences, Kampala, Uganda; 7 Department of Preventive Medicine and Community Health, New Jersey Medical School, Rutgers, The State University of New Jersey, Newark, New Jersey, United States of America; 8 Department of Microbiology & Infectious Diseases, University of Calgary, Calgary, Canada; 9 Mulago Hospital Tuberculosis Clinic, Mulago Hospital, Kampala, Uganda; 10 Division of Infectious Diseases and Global Medicine, Department of Medicine and Emerging Pathogens Institute, University of Florida, Gainesville, Florida, United States of America; Hopital Raymond Poincare - Universite Versailles St. Quentin, FRANCE

## Abstract

**Rationale:**

The diagnosis of latent tuberculosis (TB) infection (LTBI) is complicated by the absence of a gold standard. Discordance between tuberculin skin tests (TST) and interferon gamma release assays (IGRA) occurs in 10–20% of individuals, but the underlying mechanisms are poorly understood.

**Methods:**

We analyzed data from a prospective household contact study that included cough aerosol culture results from index cases, environmental and contact factors. We assessed contacts for LTBI using TST and IGRA at baseline and six weeks. We examined TST/IGRA discordance in qualitative and quantitative analyses, and used multivariable logistic regression analysis with generalized estimating equations to analyze predictors of discordance.

**Measurements and Results:**

We included 96 TB patients and 384 contacts. Discordance decreased from 15% at baseline to 8% by six weeks. In adjusted analyses, discordance was related to less crowding (p = 0.004), non-cavitary disease (OR 1.41, 95% CI: 1.02–1.96; p = 0.03), and marginally with BCG vaccination in contacts (OR 1.40, 95% CI: 0.99–1.98, p = 0.06).

**Conclusions:**

We observed significant individual variability and temporal dynamism in TST and IGRA results in household contacts of pulmonary TB cases. Discordance was associated with a less intense infectious exposure, and marginally associated with a BCG-mediated delay in IGRA conversion. Cough aerosols provide an additional dimension to the assessment of infectiousness and risk of infection in contacts.

## Introduction

Identification and treatment of latent tuberculosis (TB) infection (LTBI) is a key strategy for TB control [1]. Despite new tools and several extensive reviews on the subject [2–4], the diagnosis of LTBI in humans remains problematic. A central issue is that currently available tests have performance limitations [4–6], sub-optimal sensitivity and specificity, and they are unable to distinguish LTBI from TB disease [7]. Importantly, when intra- or inter-test disagreement arises, the final interpretation is complicated by a lack of accepted reference standard for *Mycobacterium tuberculosis* infection [5, 6, 8, 9].

Traditionally, the tuberculin skin test (TST) has been used to diagnose TB infection but it presents with multiple well-described performance and operational limitations [10]. The introduction of interferon-gamma release assays (IGRA) in clinical practice provided significant advantages over TST [2–4], but several outstanding questions remain. Firstly, there is limited information regarding the kinetics of IGRA assays. Whereas the TST is known to convert in 2–12 weeks [11, 12], the conversion interval for IGRA is not well established and may vary from 4–22 weeks [13, 14]. Some studies have suggested that IGRA becomes positive before the TST [15] but others have not [13]. Secondly, recent evidence has shown problems with the reproducibility of IGRA results [4] and the related issue of IGRA reversions (from positive to negative) [16–21]. Also, while some studies have shown that young children (<5 years of age) may be unable to mount a robust IGRA response [22, 23] others have not [4, 24]. Finally, whereas the frequency of TST/IGRA discordant results is 10%-20% in most studies [9, 25], the epidemiological and immunological basis for discordance has remained largely unexplained. Taken together, these limitations have resulted in recent calls for caution and re-interpretation of cut-offs for IGRA readouts akin to the three TST cut-off levels (5mm, 10mm and 15mm of induration) that are widely used to risk stratify individuals based on the pre-test probability of infection [6].

The absence of a gold standard for *M*. *tuberculosis* infection makes the interpretation of tests depends on the pre-test probability of infection, and therefore, knowledge of exposure is critical in assessing LTBI [5]. While acid-fast bacilli (AFB) smear microscopy has traditionally been used to risk-stratify TB exposures [26], determining the true infectiousness of pulmonary TB patients is not straightforward [27]. Our recent household study in Uganda found that cough-generated aerosols of *M*. *tuberculosis* are a much stronger predictor of recent infection (i.e. TST conversion) than sputum AFB, and observed differential TST/IGRA responses in contacts exposed to aerosol-positive vs. aerosol-negative TB patients [28]. In this secondary analysis of that study, we sought to further elucidate the observed differences in TST and IGRA responses in exposed contacts, and to examine index case, environmental and contact factors associated with TST/IGRA discordance. We hypothesized that index case cough aerosol cultures and environmental variables of exposure would help explain discordance.

## Materials and Methods

### Study population

We enrolled consecutive pulmonary TB patients attending the Mulago Hospital National Tuberculosis and Leprosy Programme (NTLP) clinic in Kampala, Uganda, and their household contacts. Eligible TB patients: 1) were ≥18 years; 2) had a new TB episode with ≥1 sputum specimen that was AFB ≥1+ with subsequent growth of *M*. *tuberculosis* in culture; 3) were untreated or had received ≤5 days of antituberculous treatment; and 4) lived with ≥3 contacts. We excluded patients with medical conditions that could be worsened by vigorous cough [29] and those who were too ill to consent or unable to comply with the protocol. A contact was an individual of any age sharing the same roof with the index TB case for ≥3 months before enrollment with no history of TB. We obtained informed consent and assent in accordance with age-specific ethical guidelines.

### Measurements

#### TB cases

Evaluation of TB cases included three sputa specimens that were digested and decontaminated using N-acetyl-L-cysteine–NaOH, and then processed for AFB smear microscopy (auramine O fluorescent stain) [30] and cultures performed on 7H11 Middlebrook agar and liquid media (MGIT 960). Radiological extent of disease was graded as normal, minimal, moderate, or far-advanced disease [31]. We evaluated the severity of cough at enrollment using a self-reported visual analog cough scale (VACS) [32, 33], three cough peak flow (CPF) measurements (MicroDirect, Inc) and subjectively, at the time of aerosol sampling (weak vs. strong). On the morning after enrollment, we cultured *M*. *tuberculosis* from cough-generated aerosols using the cough aerosol sampling system (CASS) method [28, 29]. Briefly, the CASS consists of a chamber that holds two Andersen impactors for viable bioaerosol sampling loaded with 7H11 culture plates. The number of colony forming units (CFU) of *M*. *tuberculosis* present at 6 weeks’ incubation were categorized as (1) aerosol negative; (2) low aerosol (1–9 CFU); and (3) high aerosol (≥10 CFU). We chose a 10-CFU cut-off because this was the point at which we observed increased risk in TST conversion [34]. The NTLP program provided standard TB treatment and offered routine HIV testing. HIV-infected patients had CD4 testing and were referred for HIV care.

#### Household contacts

Within two weeks of enrollment of the index TB case, we obtained demographic and clinical information in contacts, performed TST and IGRA, and visited study households to evaluate individual contact time with the index case, ventilation conditions and crowding (defined as the number of persons divided by the number of habitable rooms in the home). Following NTLP recommendations [35], contacts infected with *M*. *tuberculosis* at highest risk of developing active TB disease and secondary TB suspects were referred for treatment.

### TST and IGRA testing and retesting protocol

We trained staff in TST (Tubersol®, Sanofi Pasteur) placement and reading, and completed inter- and intra-reader evaluations (kappa >90%). We obtained blood for IGRA (Quantiferon Gold-In-Tube®, Qiagen, U.S.) testing before TST placement to avoid TST-induced IGRA boosting [36]. TST and IGRA testing were done on the same day. After collection, blood for IGRA was kept at room temperature and delivered to the laboratory on the same day, and testing was performed within 18 hrs of blood collection, as recommended by the manufacturer. The household contact evaluation was completed within 1 week after enrollment of the index TB case. A positive TST was ≥10mm. Household contacts with TST <10mm or IGRA-negative at baseline were re-tested after 6 weeks to identify TST and IGRA conversions. Baseline TST+/IGRA- were only re-tested with IGRA. TST conversion was defined as 1^st^ TST <10mm, 2^nd^ TST ≥10mm and difference between tests of ≥10mm [10]. TST/IGRA discordance was defined as having discordant results between TST (≥10mm) and IGRA either at baseline or six weeks. In order for an individual to be defined as concordant, their TST and IGRA results must have been consistent at both time points. Sensitivity analysis using alternate TST cut-off values (5 and 15 mm of induration) had an insignificant impact on the analysis of TST/IGRA discordance (data not shown).

### Statistical Methods

The study population is described using descriptive statistics stratified by discordance/concordance of TST/IGRA results. T tests, chi-square tests, Fisher exact tests, and logistic regression models using generalized estimating equations (GEE) are used to assess differences between individuals by discordance status. All attributes with a p-value <0.20 in univariate analyses were included in a multivariable logistic regression model. We retained sputum smear and aerosol (CFU) groups in the final model because of our interest in understanding how TST/IGRA results are affected by the infectious exposure, and contact age because children may have low IGRA levels [23]. Continuous measures were tested for linearity using penalized splines. We considered interactions terms that had clinical relevance. Results of this model were further explored by describing the index case characteristics and TST and IGRA results of the contacts by smear and aerosol (CFU) grouping (0, 1–9, 10+). P-values were obtained using ANOVA and logistic regression models fit with GEE. All analyses were run in SAS 9.3 (SAS Institute, Cary, NC) and R 2.15.2 (r-project.org).

### Ethical Statement

We obtained written informed consent and assent from participants in accordance with age-specific ethical guidelines from participating institutions. The study was approved by the Makerere University Faculty of Medicine Research and Ethics Committee, Uganda National Council for Science and Technology and the Institutional Review Boards of the University of Medicine and Dentistry of New Jersey (now Rutgers University) and Boston University Medical Campus.

## Results

Between May 2009 and January 2011, we enrolled 96 sputum culture-positive pulmonary TB cases and their 442 household contacts into a prospective, six-week household contact study. A detailed description of the study population and primary results of the parent study are published [34]. This secondary analysis includes only the 384 (87%) contacts with TST and IGRA results available at baseline and six weeks, per protocol. Of the 58 contacts excluded, 32 had a missing TST or IGRA results at baseline, 1 had an indeterminate IGRA result at baseline, and 25 had a missing TST or IGRA result at six weeks. Excluded contacts did not significantly differ from those studied in terms of age (p = 0.19), gender (p = 0.88), BCG scar (p = 0.81), HIV (p = 0.35), smoking (p = 1.0) or alcohol use (p = 0.35).

### TST and IGRA results in household contacts

Distribution of TST/IGRA results in household contacts at baseline and six weeks is shown in Table 1. At baseline, 18% of contacts were concordant negative (TST-/IGRA-), 67% were concordant positive (TST+/IGRA+), and 15% of contacts were discordant (i.e. TST+/IGRA- or TST-/IGRA+); by six weeks, the distribution of contacts with concordant negative, concordant positive and discordant TST/IGRA results was 12%, 80% and 8%, respectively. At both study time points, discordance was more likely to be TST+/IGRA- than TST-/IGRA+. The majority (61%) of contacts with discordance at baseline became concordant positive (TST+/IGRA+) at six weeks as a result of TST or IGRA conversions (from negative to positive); however, 16 (28%) contacts that were initially discordant remained so and 6 (11%) contacts that were initially TST-/IGRA+ reverted to concordant negative at six weeks (Table 1). Of the Of the 384 contact included in the analysis, 324 were HIV-uninfected by self-report (55 discordant, 269 concordant) and 8 were HIV-infected (1 discordant, 7 concordant); HIV status was unknown in 52 contacts.

**Table 1:** Distribution of tuberculin skin test (TST) and interferon gamma release assay (IGRA) results in household contacts at baseline and six weeks.

Study timepoint		Six weeks			
		– n (row %) –			
Baseline		TST-/IGRA-	TST+/IGRA-	TST-/IGRA+	TST+/IGRA+
– n (col %) –					
TST- / IGRA-	71 (18)	40 (56)	8 (11)	5 (7)	18 (25)
TST+ / IGRA-	31 (8)	-	10 (32)	-	21 (68)
TST- / IGRA+	26 (7)	6 (23)	-	6 (23)	14 (53)
TST+ / IGRA+	256 (67)	-	-	-	256 (100)
Totals	384 (100)	46 (12)	18 (5)	11 (3)	309 (80)

**Fig 1:**
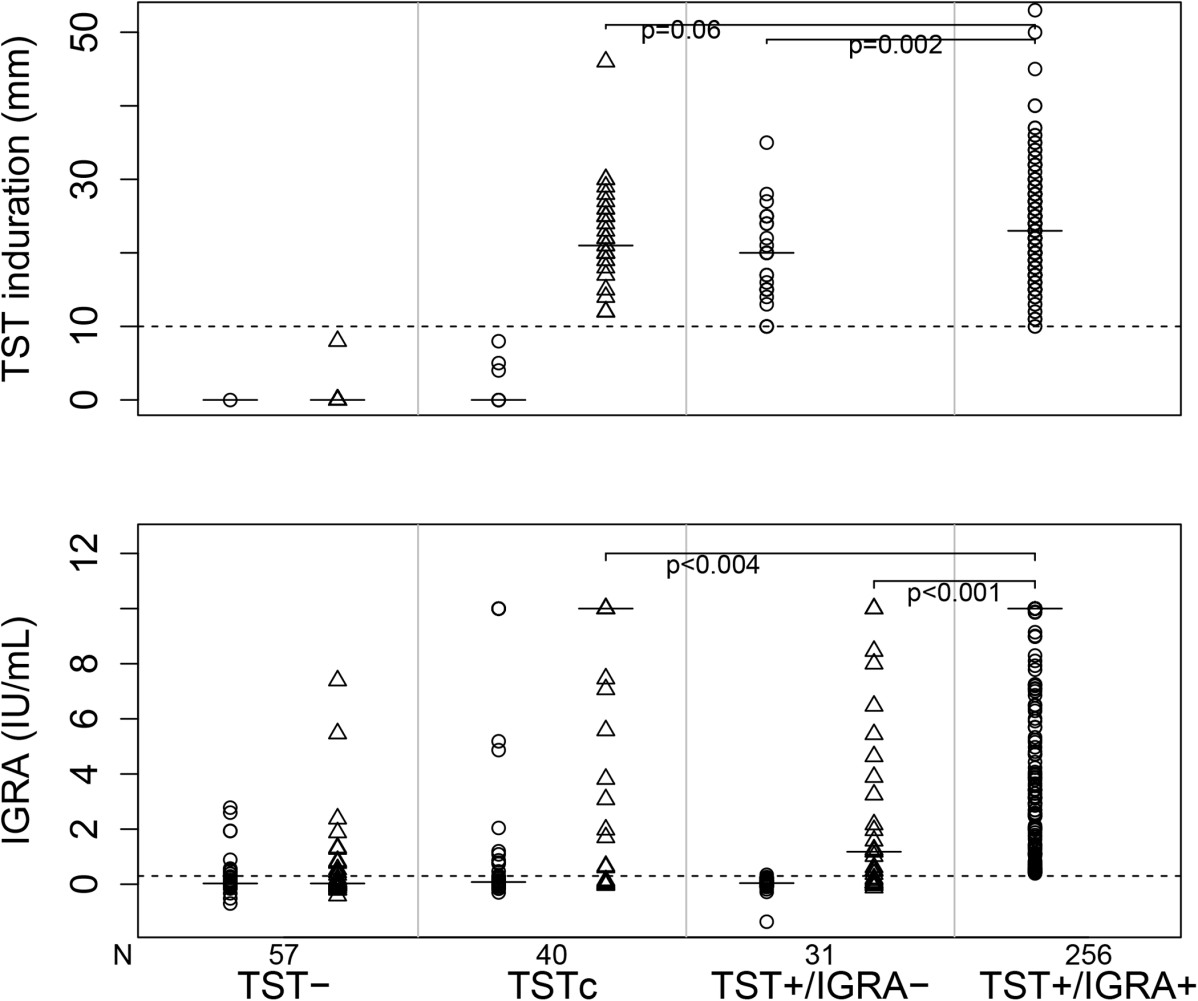
Quantitative analysis of tuberculin skin test (TST) and interferon gamma release assay (IGRA) results among four groups of contacts exposed to a patient with pulmonary tuberculosis: 1) TST- = contacts who have TST<10mm at baseline and 6 weeks; 2) TSTc = contacts who undergo TST conversion at 6 weeks (see definition in *Methods*); 3) TST+/IGRA- = contacts with TST ≥10mm at baseline and IGRA-; and 4) TST+/IGRA+ = contacts with TST ≥10mm at baseline and IGRA+. Circles indicate baseline values and triangles indicate values at 6 weeks. The solid horizontal bars indicate the median value. The N for each group is the same for TST and IGRA results.

To further understand the nature of discordance, we performed a quantitative analysis of TST/IGRA results in contacts according to four TST-defined, clinically-relevant groups: 1) TST-negative at both baseline and six weeks; 2) TST-converters, and; 3) TST-positive at baseline/IGRA-negative; and 4) TST-positive at baseline/IGRA-positive. There was significant variation in quantitative IGRA readouts across groups, both among TST-negative and TST-positive individuals (Fig 1). Six-week IGRA readouts in contacts that were TST+/IGRA- at baseline were significantly lower (median: 1.18 IU/mL, range: -0.12–10.00 IU/mL) when compared to contacts that were TST+/IGRA+ at baseline (10.00 IU/mL, 0.39–10.00 IU/mL, p<0.001) and TST-converters (10.00 IU/mL, -0.03–10.00 IU/mL; p = 0.004); similarly, the six-week median TST diameter in contacts that were TST+/IGRA- at baseline was smaller (20 mm, 10-35mm) when compared to the other two groups but to a lesser degree (23 mm, 10-53mm; p = 0.002, and 21 mm, 12-46mm; p = 0.06, respectively) (Fig 1).

### Factors associated with TST/IGRA discordance

We then sought to identify index case, environmental and contact characteristics associated with TST/IGRA discordance (Table 2 and S1 Table).

**Table 2:** Univariate and multivariable analyses of factors associated with TST/IGRA discordance in household contacts.

	Household Contacts							
Characteristic	Overall	Concordant	Discordant		Unadjusted Odds Ratio	*P*-value	Adjusted^1^ Odds Ratio	*P*-value
	N = 384	N = 314	N = 70		(95% Confidence Interval)		(95% Confidence Interval)	
Index cases								
Age (per 5-year increment)	31.80 [11.54]	31.83 [11.61]		31.69 [11.32]	1.05 (0.90–1.22)	0.73		
Sex								
Male	184 (48)	150 (48)		34 (49)	1.09 (0.55–2.18)	0.80		
Female	200 (52)	164 (52)		36 (51)	REF			
Cough measurements at baseline								
Visual analog cough scale^2^	4.54 [1.54]	4.61 [1.51]		4.23 [1.60]	0.84 (0.66–1.06)	0.13	0.75 (0.53–1.06)	0.10
Cough peak flow^3^	242.63 [85.37]	242.73 [82.16]		242.14 [99.17]	1.00 (0.99–1.00)	0.84		
Cough during aerosol collection						0.97		
Weak	157 (41)	128 (41)		29 (41)	0.99 (0.50–1.99)			
Strong	227 (59)	186 (59)		41 (59)	REF			
Chest radiograph								
Extent of lung disease						0.34		
Normal/Minimal	37 (10)	26 (9)		11 (16)	2.47 (0.72–8.47)			
Moderate	120 (31)	94 (32)		26 (38)	1.49 (0.71–3.13)			
Far advanced	205 (53)	174 (59)		31 (644)	REF			
Cavitations						0.05		
Absent	124 (32)	91 (31)		33 (49)	2.10 (1.04–4.21)		1.41 (1.02–1.96)	0.03
Present	238 (62)	202 (69)		35 (51)	REF		REF	
Sputum AFB smear						0.56		0.31
Negative/ Scanty/ 1+	68 (18)	52 (17)		16 (23)	1.45 (0.66–3.17)		1.33 (0.74–2.39)	
2+	62 (16)	48 (15)		14 (20)	1.55 (0.57–4.22)		1.10 (0.52–2.33)	
3+	254 (66)	214 (68)		40 (57)	REF		REF	
7H11 culture (CFU)						0.26		0.20
<200	61 (16)	46 (15)		15 (21)	1.66 (0.75–3.68)		1.40 (0.86–2.26)	
≥200	323 (84)	268 (85)		55 (79)	REF		REF	
Cough aerosol (CFU)						0.49		
Negative	217 (57)	177 (56)		40 (57)	1.47 (0.66–3.28)			
Low (1–9)	79 (21)	62 (20)		17 (24)	1.76 (0.64–4.85)			
High (≥10)	88 (23)	75 (24)		13 (19)	REF			
**Household contacts**								
Age (per 5-ear increment)	18.37 [14.50]	18.41 [14.02]		18.19 [16.47]	0.95 (0.86–1.05)	0.40		
Sex						0.22		0.15
Male	165 (43)	130 (41)		35 (50)	1.41 (0.84–2.36)		1.26 (0.92–1.72)	
Female	219 (57)	184 (59)		35 (50)	REF		REF	
BCG scar						0.11		0.06
Absent/uncertain	91 (24)	79 (25)		12 (18)	REF		REF	
Present	288 (76)	232 (75)		56 (82)	1.71 (0.85–3.43)		1.40 (0.99–1.98)	
Sleeping arrangement with index						0.01		0.17
Different room	185 (48)	145 (46)		40 (57)	3.19 (1.21–8.40)		1.51 (0.89–2.55)	
Same room, different bed	146 (38)	120 (38)		26 (37)	2.47 (1.08–5.66)		1.15 (0.66–2.02)	
Same room, same bed	53 (14)	49 (16)		4 (6)	REF		REF	
**Dwellings**								
Crowding measure						0.02		0.004
<1 contact/room	62 (17)	39 (13)		23 (33)	4.72 (1.87–11.90)		2.88 (1.54–5.37)	
1–2 contacts/room	129 (35)	101 (34)		28 (41)	2.36 (1.02–5.48)		1.69 (1.02–2.79)	
2–3 contacts/room	93 (25)	85 (28)		8 (12)	0.87 (0.27–2.75)		0.53 (0.25–1.09)	
>3 contacts/room	86 (23)	76 (25)		10 (14)	REF		REF	

Values are mean [Standard deviation] or n (percent), unless otherwise specified.

P-values are calculated using a logistic regression model fit with generalized estimating equations (GEE) approach.

AFB = Acid fast-bacilli; CFU = Colony forming units of *M*. *tuberculosis* in aerosols.

Missing data: Index case HIV status (3), chest radiograph (22, 20 concordant and 2 discordant), contact HIV history (52), and meals shared with index case (1).

^1^Univariate and multivariable model adjusted for correlation between contacts with the same index case using a GEE model. The final adjusted model only uses those that had p<0.2 in the univariate results + CASS and smear.

^2^ Visual analog scale is per unit increase.

^3^ Cough peak flow is per 10 L/min increase.

*Index case factors*: The absence of cavitations on the chest radiograph was associated with discordance both in the unadjusted analysis (p = 0.05) and the multivariable model (odds ratio [OR] 1.41, 95% confidence interval (CI): 1.02–1.96; p = 0.03). A weaker cough (OR = 0.75 for every unit increase in VACS, 95% CI: 0.53–1.06; p = 0.10) was marginally associated with discordance in the multivariable model.

*Environmental factors*: Discordance was associated with less household crowding in both the univariate analysis (p = 0.02) and in the multivariable model (<1 contact/room OR 2.88, 95% CI: 1.54–5.37; and 1–2 contacts/room OR 1.69, 95% CI: 1.02–2.79 vs. > 3 contacts/room; overall p = 0.004).

*Household contact factors*: Discordance was marginally associated with presence of a BCG scar in both the univariate analysis (p = 0.11) and the multivariable model (OR = 1.40, 95% CI: 0.99–1.98; p = 0.06). As TST boosting may occur from BCG vaccination, particularly in children under 10 years of age [37], we evaluated the interaction between age and BCG, but it was not significant, but possibly underpowered, in our multivariable model (p = 0.16) (Table 3). At baseline, the proportion of contacts with TST ≥10mm or IGRA positive increased from 72.4% in contacts age ≤10 years to 85.7% in those >10 years. However, in contacts ≤10 years the frequency of TST/IGRA discordance in BCG-vaccinated contacts (19%) was nearly doubled compared to those with no BCG scar (10%, p = 0.24); we observed a similar result (16% vs. 7%, p = 0.076) in older contacts.

**Table 3:** Baseline tuberculin skin test (TST) and interferon gamma release assay (IGRA) results by BCG vaccination scar^*^ and contact age.

	≤10 years old		>10 years old	
	BCG scar	No BCG scar	BCG scar	No BCG scar
TST-/IGRA-	26 (27)	9 (29)	27 (14)	9 (15)
TST+/IGRA-	9 (9)	1 (3)	17 (9)	2 (3)
TST-/IGRA+	9 (9)	2 (6)	13 (7)	2 (3)
TST+/IGRA+	52 (54)	19 (61)	135 (70)	47 (78)
Total	96	31	192	60

* Five contacts had uncertain BCG scar results

### Infectiousness of index TB patients: sputum vs. aerosol results

In light of the above results, we investigated the use of sputum vs. aerosols to risk stratify the infectious exposure. Table 4 (upper section) shows index TB case characteristics according to sputum AFB grade and aerosol CFUs. Sputum AFB smear microscopy grading was associated with several measures of disease severity (Table 4, upper section) such as length of illness (p = 0.04), sputum semi-quantitative Middlebrook (p = 0.01) and MGIT (p = 0.04) culture results; and, weakly correlated with sputum volume (p = 0.06) and age (p = 0.07). In contrast, aerosol CFU (0, 1–9 and ≥10) was not associated with any of these measures of TB disease progression or bacillary burden but was correlated with cough severity (p = 0.01), as measured by cough peak flow. The majority (254, 66.1%) of contacts were exposed to sputum AFB 3+ patients, but only a minority (88, 22.9%) was exposed to high-aerosol TB patients.

**Table 4:** Characteristics of index cases and household contacts and of tuberculin skin test (TST) and interferon gamma release assay (IGRA) results in contacts. Results are shown by sputum acid-fast bacilli (AFB) smear microscopy grade and colony forming units (CFU) of *M*. *tuberculosis* cultured in aerosols.

Characteristic	Overall	Sputum (AFB)				Aerosol (CFU)			
		1+*	2+	3+	P Value^+^	0	1–9	≥10	P value^+^
Index cases	N = 96	16	14	66		53	18	25	
Age (years)	28.9	26.9	29.4	30.0	0.07	29.3	28.4	28.5	0.98
	[22.8–39.2]	[21.5–30.1]	[20.4–40.1]	[24.0–40.3]		[23.1–36.5]	[20.8–40.3]	[24.0–40.0]	
Sex					0.12				0.55
Male	49 (51)	7/16 (44)	4/14 (29)	38/66 (58)		26/53 (49)	8/18 (44)	15/25 (60)	
Female	47 (49)	9/16 (56)	10/14 (71)	28/66 (42)		27/53 (51)	10/18 (56)	10/25 (40)	
Weeks sick before enrollment	12.0 [8.0–16.0]	8.0 [3.5–14.0]	12.0 [8.0–16.0]	12.0 [8.0–20.0]	0.04	12.0 [8.0–12.0]	12.0 [8.0–12.0]	12.0 [8.0–15.0]	0.39
Cough measurements:									
VACS^1^	4.0 [4.0–6.0]	4.0 [4.0–5.0]	4.5 [3.0–5.0]	4.0 [4.0–6.0]	0.76	4.0 [4.0–6.0]	4.0 [3.0–5.0]	4.5 [4.0–5.0]	0.45
Cough peak flow^2^	200 [130–250]	225 [120–305]	200 [140–230]	195 [130–250]	0.58	190 [120–220]	180 [140–270]	250 [190–300]	0.01
Cough during CASS					0.54				0.49
Strong	60 (63)	11 (69)	7 (50)	42 (64)		32 (60)	10 (56)	18 (72)	
Weak	36 (37)	5 (31)	7 (50)	24 (36)		21 (40)	8 (44)	7 (28)	
Sputum volume (mL)	5.0 [4.0–10.0]	4.5 [2.3–7.0]	3.5 [3.0–5.0]	5.0 [5.0–10.0]	0.06	5.0 [4.0–10.0]	5.0 [3.0–7.5]	5.0 [5.0–15.0]	0.24
*Chest radiograph*:									
Extent of lung disease					0.26				0.47
Normal/Minimal	8 (9)	2 (15)	0 (0)	6 (9)		4 (8)	3 (19)	1 (4)	
Moderate	30 (33)	6 (46)	6 (46)	18 (28)		15 (29)	6 (38)	9 (38)	
Far advanced	53 (58)	5 (39)	7 (54)	41 (63)		32 (63)	7 (43)	14 (58)	
Cavitation					0.60				0.25
Absent	30 (33)	6 (46)	4 (31)	20 (31)		14 (27)	8 (50)	8 (33)	
Present	61 (67)	7 (54)	9 (69)	45 (69)		37 (73)	8 (50)	16 (67)	
Sputum 7H11 culture (CFU)					0.01				0.89
<20	6 (6)	2 (12)	0 (0)	4 (6)		5 (9)	0 (0)	1 (4)	
20–99	4 (4)	2 (12)	1 (7)	1 (2)		2 (4)	1 (6)	1 (4)	
100–199	3 (3)	2 (12)	1 (7)	0 (0)		2 (4)	0 (0)	1 (4)	
≥200	83 (87)	10 (64)	12 (86)	61 (92)		44 (83)	17 (94)	22 (88)	
Sputum MGIT culture (DTP)	6 [4–8]	9 [7–11]	7 [7–9]	5 [3–6]	0.04	6 [4–8]	5 [4–8]	6 [4–10]	0.69
Household contacts—N	384	68	62	254	P-value^^^	217	79	88	P-value^^^
Age (years)					0.24				0.54
Overall	14 [8–25]	18 [9–28]	14 [7–26]	14 [8–23]		14 [8–24]	15 [9–24]	14 [7–27]	
≤5 years	63 (16)	8 (12)	12 (19)	43 (17)		31 (14)	13 (16)	19 (22)	
6–15 years	152 (39)	24 (35)	26 (42)	102 (40)		92 (42)	32 (41)	28 (32)	
≥16 years	169 (44)	36 (53)	24 (39)	109 (43)		94 (43)	34 (43)	41 (47)	
BCG scar					0.56				0.98
Present	288 (75)	50 (74)	43 (69)	195 (77)		163 (75)	58 (73)	67 (76)	
Absent	91 (24)	17 (25)	19 (31)	55 (22)		50 (23)	20 (25)	21 (24)	
Uncertain	5 (1)	1 (1)	0 (0)	4 (2)		4 (2)	1 (1)	0 (0)	
TST ≥10 mm at baseline									
Overall	287/384 (75)	49/68 (72)	38/62 (61)	200/254 (79)	0.27	163/217 (75)	54/79 (68)	70/88 (80)	0.27
≤5 years	42/63 (67)	5/8 (63)	9/12 (75)	28/43 (65)	0.97	21/31 (68)	7/13 (54)	14/19 (74)	0.76
6–15 years	106/152 (70)	13/24 (54)	14/26 (54)	79/102 (77)	0.08	64/92 (70)	22/32 (69)	20/28 (71)	0.87
≥16 years	139/169 (82)	31/36 (86)	15/24 (63)	93/109 (85)	0.21	78/94 (83)	25/34 (74)	36/41 (88)	0.25
IGRA+ at baseline									
Overall	282/384 (73)	48/68 (71)	39/62 (63)	195/254 (77)	0.28	162/217 (75)	54/79 (68)	66/88 (75)	0.60
≤5 years	40/63 (63)	3/8 (38)	10/12 (83)	27/43 (63)	0.29	20/31 (65)	7/13 (54)	13/19 (68)	0.81
6–15 years	106/152 (70)	15/24 (63)	13/26 (50)	78/102 (76)	0.12	65/92 (71)	21/32 (66)	20/28 (71)	0.87
≥16 years	136/169 (80)	30/36 (83)	16/24 (67)	90/109 (83)	0.33	77/94 (82)	26/34 (76)	33/41 (80)	0.83
TST conversion ^3^									
Overall	39/97 (40)	10/19 (53)	6/24 (25)	23/54 (43)	0.53	17/54 (31)	7/25 (28)	15/18 (83)	0.04
≤5 years	8/21 (38)	2/3 (67)	2/3 (67)	4/15 (27)	0.31	3/10 (30)	1/6 (17)	4/5 (80)	0.10
6–15 years	22/46 (48)	5/11 (45)	2/12 (17)	15/23 (65)	0.16	11/28 (39)	4/10 (40)	7/8 (88)	0.12
≥16 years	9/30 (30)	3/5 (60)	2/9 (22)	4/16 (25)	0.54	3/16 (19)	2/9 (22)	4/5 (80)	0.14
IGRA conversion ^3^									
Overall	44/102 (43)	8/20 (40)	9/23 (39)	27/59 (46)	0.57	17/55 (31)	10/25 (40)	17/22 (77)	0.01
≤5	6/23 (26)	1/5 (20)	2/2 (100)	3/16 (19)	N/A	4/11 (36)	0/6 (0)	2/6 (33)	N/A
6–15	19/46 (41)	3/9 (33)	4/13 (31)	12/24 (50)	0.68	8/27 (30)	4/11 (36)	7/8 (88)	0.07
≥16	19/33 (58)	4/6 (67)	3/8 (38)	12/19 (63)	0.40	5/17 (29)	6/8 (75)	8/8 (100)	N/A

Values are median [IQR] or n (%), unless otherwise specified.

^*^ Group includes one patient with smear = negative and one smear = scanty.

^+^P-values for index case factors obtained using Fisher exact test; ^P-values for contact factors obtained using GEE models.

AFB = Acid fast-bacilli; CFU = Colony forming units of *M*. *tuberculosis* in aerosols; VACS = Visual analog cough scale; CASS = Cough aerosol sampling system; and DTP = Days to positive.

^1^ Visual analog cough scale is per unit increase.

^2^ Cough peak flow is per 10 L/min increase.

Missing data for index TB cases: Week sick before enrollment (1), Extent of lung disease/cavitation (5).

Missing data for household contacts: Age (2).

^3^ Only includes contacts “at risk” of TST (<10mm at baseline) or IGRA (negative at baseline) conversion.

### TST and IGRA results according to sputum vs. aerosol

**Fig 2:**
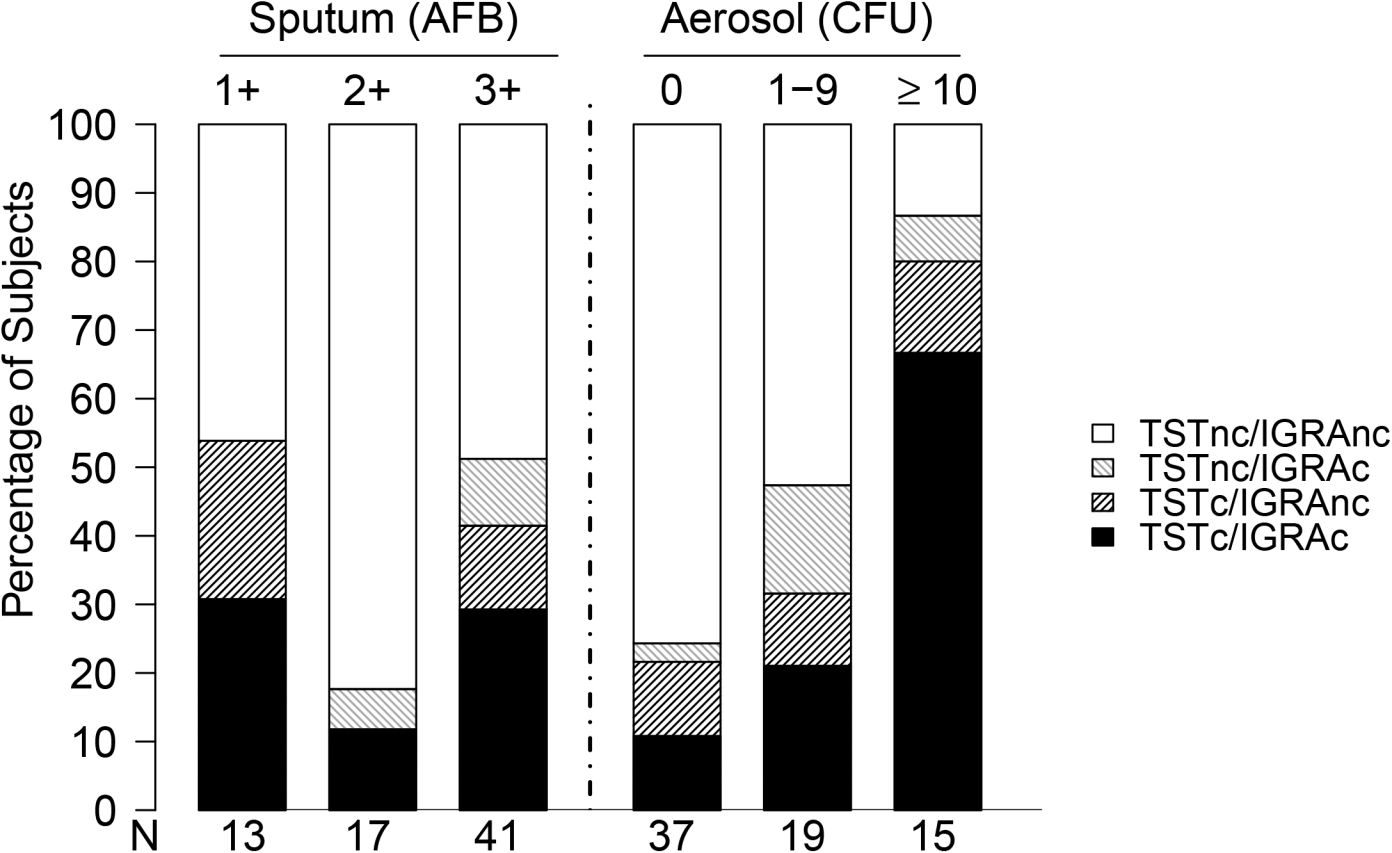
Analysis of tuberculin skin test (TST) and interferon gamma release assay (IGRA) readouts at six weeks among contacts TST and/or IGRA negative at baseline by exposure variables measured by AFB smear and CASS aerosol CFU. “c” = conversion (from negative to positive); “nc” = non-conversion

We analyzed prevalent and incident TST/IGRA results according to exposure category (i.e. sputum vs. aerosol) and contact age (Table 4, lower section). At baseline, the overall prevalence of TST (75%) and IGRA positivity (73%) were similar, and did not significantly differ by exposure (both p = 0.27). A quantitative analysis of TST and IGRA readouts at baseline showed similar results (S2 Table). At 6 weeks, there was a significant dose-response association between aerosol CFU and TST (p = 0.04) or IGRA (p = 0.01) conversion, but using sputum AFB smear grade to classify exposure groups did not show distinct risk stratification (p>0.50). We observed a similar dose-response relationship between aerosol CFU and incident *M*. *tuberculosis* infection with combined TST and IGRA results (Fig 2); however, the frequency, distribution and type (TST+/IGRA- vs. TST-/IGRA+) of discordance was similar across exposure groups. The quantitative analysis of TST and IGRA readouts was limited by small cell numbers (S3 Table).

## Discussion

In this Ugandan household contact study we found significant inter-subject variability and dynamism in TST/IGRA results. Our findings suggest that TST/IGRA discordance may be largely explained by differences in the lag interval for achieving high sensitivity between these two tests used to diagnose LTBI–most often manifested by a delay in IGRA conversion. Discordance was more frequent in contacts of pulmonary TB patients with less advanced disease and in those living in less crowded dwellings where physical proximity may be reduced. We suggest that in these individuals the likelihood for *M*. *tuberculosis* infection to occur after study initiation is increased compared to contacts of TB patients with more advanced disease, or living in densely crowded households who were likely to be infected earlier (e.g. prevalent at baseline). Discordance was also associated with BCG vaccination in contacts, an interesting finding that will need to be examined in future studies. However, these data did not support our hypothesis that cough aerosol cultures from the index case, as a measure of source strength and inhaled dose, are associated with discordance.

Since their introduction in clinical practice, IGRA tests have shown significant disagreement with TST results [25]. Early evidence–mainly from cross-sectional studies conducted in low TB prevalence settings- attributed much of the observed discordance to false-positive TST results (mostly ascribed to BCG vaccination), heralding IGRA testing as a strategy to improve the efficiency of LTBI programs [25, 38–42]. More recently, there is increasing recognition that IGRA readouts are less reproducible than initially thought, with significant within-subject variability [6, 19, 36, 43–45] and unusual measurements [46]. Serial testing with IGRAs in low risk settings (U.S. and Canada) has shown high IGRA conversion rates (4–7%) when compared to historical or concurrent TST conversions rates (0.0–0.9%) [16–18]; notably, 60–75% of individuals with IGRA conversion in these studies reverted to IGRA negative upon repeat testing. Unusually high rates of IGRA conversions and reversions have also been reported in high TB prevalence settings [20, 21, 47]. In our current study, 23% of contacts with TST-/IGRA+ discordance at baseline reverted to concordant negative upon further testing. Taken together, these data are reminiscent of the known instability of TST across populations with differing risk for *M*. *tuberculosis* infection [10, 12, 48, 49]. Several sources of variability (manufacturing, pre-analytical, analytical and immunological) have been identified that may impact the reproducibility of IGRA results [4]. The results from this study and others [47, 50] suggest that a delay in IGRA conversion should be added to the possible causes of discordant TST/IGRA results in recently exposed contacts.

Taken together, the variability and dynamism in TST/IGRA results we observed in both this and our parent study [34], highlight the value of interpreting *M*. *tuberculosis* transmission and infection outcomes through the prism of aerosol CFU, rather than sputum AFB. Whereas a single positive TST or IGRA result cannot distinguish contacts with LTBI from those with prevalent or incident active disease [7, 51, 52], the value of TST size to predict risk of progression to disease is well established [53, 54], and there is a growing body of evidence showing quantitative IGRA readouts are associated with increased risk of disease [7, 51, 55, 56]. Also, contacts that are double positive (TST+ and IGRA+) may be at greater risk of progression to disease than those who are TST- or IGRA-positive alone [7]. Contacts of high-aerosol producing patients shared all of these measures of risk including higher rates of concomitant TST and IGRA conversion, larger TST diameters, and larger IGRA readouts compared to other contacts. These findings underscore the potential importance of bacterial viability and the associated issue of inoculum size at the time of infection in humans–a notion that is consistent with a large body of data demonstrating the role of infecting dose in determining immunopathology and survival in experimental conditions [57–59].

The role of BCG vaccination in interpreting LTBI diagnostic tests remains an area of uncertainty. BCG vaccination at birth is considered to have a negligible impact on TST results 10 years post-vaccination [4, 37]. In this study we did not see a differential impact of BCG vaccination by age on TST results nor in discordance between the TST and IGRA results. However, we found evidence of a potential effect of BCG vaccination and TST/IGRA discordance–predominantly in the form of TST+/IGRA- results. The frequency of discordance between BCG-vaccinated and unvaccinated individuals was similar among younger (<10 years) and older (≥10 years) contacts, making this unlikely to be due to false-positive TST results. We suggest that it is more likely that BCG vaccination is delaying (or preventing) IGRA conversion in newly infected contacts. Although still controversial, we [50, 60] and others [61–63] have found similar evidence of an interaction between BCG and IGRA results.

This study emphasizes how exposure impacts household transmission dynamics. Our results stratified by aerosol CFUs, a more refined predictor of transmission and inhaled dose than sputum, lead to the following observations: 1) Sputum AFB grading progresses linearly as pulmonary disease severity advances over time (Table 4, upper section); in contrast, aerosol CFU is unrelated to markers of TB disease progression or bacterial load; 2) The hazard of infection for sputum AFB follows a U-shape function as the highest risk of infection is in contacts exposed to 1+ and 3+ patients; we speculate the former may be due to a larger number of uninfected contacts “at risk” of new infection (contact factor) while the latter is driven by exposure time and increased infectiousness in the TB case as disease progresses (index case factor). With aerosols, the infection hazard follows a standard dose-response curve, of increasing risk of infection with aerosol CFU numbers (e.g. index case factor alone); 3) The frequency and type of discordance is similarly distributed across exposure groups (sputum vs. aerosol), suggesting discordance is unrelated to inoculum size and, more likely due to idiosyncratic host immune responses in the infected contact, and; 4) Clinically indistinguishable infectious exposures lead to both qualitative and quantitative differences in TST and IGRA readouts. While the epidemiologic and immunologic basis for this variability are unknown at this time, the dose-response distribution of TST/IGRA readouts across aerosol categories suggest that any positive signal in TST or IGRA readouts is indicative of infection. Taken together, our results suggest the assessment of LTBI should include both a TST and IGRA as they provide complimentary value, particularly when coupled with cough-aerosol results.

This study has some limitations. First, most of our conclusions are based on observed incident infections (i.e. contacts that become infected after study initiation). Whereas the inclusion of prevalent infections would provide a more complete assessment, these constitute a mix of old (community) exposures and recent infections (exposure to index case) that occur in the period immediately before study. In this setting, we estimate ~25–30% of prevalent infections are old while ~40–45% result from the index case exposure [64], but current technologies are unable to distinguish between them, and thus, their inclusion would significantly confound results. Also, while important, our analyses of quantitative TST/IGRA readouts and BCG-interaction were limited by small numbers. Contacts were not tested for HIV and thus, HIV infection was not taken into account in interpreting TST/IGRA results.

In conclusion, the interpretation of LTBI results is complicated by the lack of a reference method, imperfect tests and a rapidly evolving literature. The results from this study confirm that knowledge of exposure is key to interpreting LTBI results. BCG vaccination may be partially responsible for TST/IGRA discordance by delaying (or abrogating) IGRA conversion. Aerosol measurements provide an additional dimension to the assessment of infectiousness and risk of infection in contacts. Their use in future studies may help elucidate TB immunopathogenesis and predict the development of disease among infected individuals.

## Supporting Information

S1 Table Univariate and multivariable analyses of factors (additional factors from Table 2) associated with TST/IGRA discordance in household contacts. Legend: Values are mean [Standard deviation] or n (percent), unless otherwise specified P-values are calculated using a logistic regression model fit with generalized estimating equations (GEE) approach. AFB = Acid fast-bacilli; CFU = Colony forming units of *M*. *tuberculosis* in aerosols; DTP = Days to positive (per 1 day). Missing data: Index case HIV status (3), chest radiograph (22, 20 concordant and 2 discordant), contact HIV history (52,) and meals shared with index case (1). ^1^ None of the variables included in this table were adjusted (all p>0.2). ^2^ Visual analog scale odds ratio is per unit increase. ^3^ Cough peak flow odds ratio is per 10 L/min increase.
(DOCX)

S2 Table Quantitative analysis of tuberculin skin test (TST) and interferon gamma release assay (IGRA) results at baseline (prevalent infection) among exposed contacts according to exposure variable. Legend: Values are median [interquartile range] or n (percent). ^*^ Group includes one patient with smear = negative and one smear = scanty. AFB = Acid fast-bacilli; CFU = Colony forming units of *M*. *tuberculosis* in aerosols. IGRA values are capped at 10. Those with values greater than 10 are reported as >10 for this analysis.
(DOCX)

S3 Table Analysis of quantitative tuberculin skin test (TST) and interferon gamma release assay (IGRA) readouts at six weeks among contacts “at risk” for new *M*. *tuberculosis* infection (i.e. TST and/or IGRA negative at baseline) by exposure variable. Legend: Values are median [interquartile range] or n (percent) ^*^ Group includes one patient with smear = negative and one smear = scanty AFB = Acid fast-bacilli; CFU = Colony forming units of *M*. *tuberculosis* in aerosols IGRA values are capped at 10 IU/mL. Those with values greater than 10, are given the value >10 for this analysis. “c” = conversion (from negative to positive); “nc” = non-conversion.
(DOCX)
