# Supplementary material for: Cough Aerosol Cultures of Mycobacterium tuberculosis: Insights on TST / IGRA Discordance and Transmission Dynamics
Source: PLoS One. 2015 Sep 22;10(9):e0138358. doi: 10.1371/journal.pone.0138358 (PMC4578948; doi:10.1371/journal.pone.0138358)
Supplement: S1 Table — Legend: Values are mean [Standard deviation] or n (percent), unless otherwise specified P-values are calculated using a logistic regression model fit with generalized estimating equations (GEE) approach. AFB = Acid fast-bacilli; CFU = Colony forming units of M. tuberculosis in aerosols; DTP = Days to positive (per 1 day). Missing data: Index case HIV status (3), chest radiograph (22, 20 concordant and 2 discordant), contact HIV history (52,) and meals shared with index case (1). 1 None of the variables included in this table were adjusted (all p>0.2). 2 Visual analog scale odds ratio is per unit increase. 3 Cough peak flow odds ratio is per 10 L/min increase. (DOCX) [file pone.0138358.s001.docx]

**Table S1: Univariate and multivariable analyses of factors (additional factors from Table 2) associated with TST/IGRA discordance in household contacts.**

| **Characteristic** | **Household Contacts** | | |  | |  |  |
| --- | --- | --- | --- | --- | --- | --- | --- |
|  | **Overall**  **N=384** | **Concordant**  **N=313** | **Discordant**  **N=71** | **Unadjusted Odds Ratio**  **(95% Confidence Interval)** | ***P*-value** | **Adjusted^1^ Odds Ratio**  **(95% Confidence Interval)** | ***P*-value** |
| **Index cases** |  |  |  |  |  |  |  |
| Time sick prior to enrollment (weeks) | 13.8 [7.7] | 14.0 [11.6] | 13.0 [8.6] | 0.98 (0.92-1.04) | 0.55 |  |  |
| HIV status  Infected  Uninfected  Missing | 84 (22)  297 (77)  3 (1) | 67 (21)  243 (78)  3 (1) | 17 (24)  54 (76)  0 (0) | REF  1.47 (0.65-3.29) | 0.35 |  |  |
| Sputum volume (per 1 ml) | 7.3 (6.5) | 7.6 [6.6] | 6.3 [6.2] | 0.99 (0.92-1.07) | 0.82 |  |  |
| MGIT 960 (DTP) | 6.4 [4.3] | 6.3 [4.6] | 6.9 [2.9] | 1.01 (0.95-1.08) | 0.69 |  |  |
| **Household contacts** |  |  |  |  |  |  |  |
| History of HIV  Yes  No | 8 (2)  324 (85) | 7 (2)  269 (86) | 1 (1)  55 (77) | REF  1.30 (0.19-8.80) | 0.78 |  |  |
| Meals shared with index case  0  1  2  3+ | 10 (2.6)  127 (33)  160 (42)  86 (22) | 8 (3)  102 (33)  137 (44)  65 (21) | 2 (3)  25 (35)  23 (32)  21 (30) | REF  1.89 (0.61-5.84)  1.35 (0.52-3.50)  2.14 (0.66-6.93) | 0.33 |  |  |
| **Dwellings** |  |  |  |  |  |  |  |
| Daily contact time with index case  <1 hour  1-6 hours  7-12 hours  13-18 hours  >18 hours | 4 (1)  17 (4)  214 (56)  65 (17)  84 (22) | 3 (1)  14 (4)  177 (57)  57 (18)  62 (20) | 1 (0)  3 (4)  37 (52)  8 (11)  22 (31) | 0.49 (0.20-1.19)  0.62 (0.20-1.95)  0.96 (0.38-1.25)  0.50 (0.23-1.09)  REF | 0.36 |  |  |

Values are mean [Standard deviation] or n (percent), unless otherwise specified

P-values are calculated using a logistic regression model fit with generalized estimating equations (GEE) approach.

AFB= Acid fast-bacilli; CFU= Colony forming units of *M. tuberculosis* in aerosols; DTP= Days to positive (per 1 day).

Missing data: Index case HIV status (3), chest radiograph (22, 20 concordant and 2 discordant), contact HIV history (52, ) and meals shared with index case (1).

^1^ None of the variables included in this table were adjusted (all p>0.2).

^2^ Visual analog scale odds ratio is per unit increase.

^3^ Cough peak flow odds ratio is per 10 L/min increase.
