# Supplementary material for: Cough Aerosol Cultures of Mycobacterium tuberculosis: Insights on TST / IGRA Discordance and Transmission Dynamics
Source: PLoS One. 2015 Sep 22;10(9):e0138358. doi: 10.1371/journal.pone.0138358 (PMC4578948; doi:10.1371/journal.pone.0138358)
Supplement: S2 Table — Legend: Values are median [interquartile range] or n (percent). * Group includes one patient with smear = negative and one smear = scanty. AFB = Acid fast-bacilli; CFU = Colony forming units of M. tuberculosis in aerosols. IGRA values are capped at 10. Those with values greater than 10 are reported as >10 for this analysis. (DOCX) [file pone.0138358.s002.docx]

**Table S2: Quantitative analysis of tuberculin skin test (TST) and interferon gamma release assay (IGRA) results at baseline (prevalent infection) among exposed contacts according to exposure variable.**

| Index case exposure category | Overall | Sputum (AFB) | | | | Aerosol (CFU) | | | |
| --- | --- | --- | --- | --- | --- | --- | --- | --- | --- |
|  |  | 1+* | 2+ | 3+ | P value | 0 | 1-9 | ≥10 | P value |
| Contacts - N | 384 | 68 | 62 | 254 |  | 217 | 79 | 88 |  |
| **TST-/IGRA-**  n (%)  TST (mm)  IGRA (IU/mL) | 71 (18)  0 [0-0]  0.01  [-0.03-0.07] | 13 (19)  0 [0-0]  0.02  [-0.01-0.05] | 17 (27)  0 [0-0]  -0.02  [-0.11-0.03] | 41 (16)  0 [0-0]  0.02  [0-0.12] | N/A  0.09 | 37 (17)  0 [0-0]  0  [-0.06-0.04] | 19 (24)  0 [0-0]  0.02  [0-0.16] | 15 (17)  0[0-0]  0.02  [-0.03-0.08] | N/A  0.11 |
| **TST+/IGRA-**  n (%)  TST (mm)  IGRA (IU/mL) | 31 (8)  20 [15-24]  0.04  [-0.05-0.15] | 7 (10)  20 [14-24]  0.05  [0-0.14] | 6 (10)  20.5 [20-25]  0.075  [-0.09-0.23] | 18 (7)  18.5 [15-24]  0.03  [-0.05-0.19] | 0.47  N/A | 18 (8)  20 [16-25]  0.03  [-0.05-0.14] | 6 (8)  15 [15-20]  0.03  [-0.18-0.23] | 7 (8)  20 [14-25]  0.19  [0-0.26] | 0.17  N/A |
| **TST-/IGRA+**  n (%)  TST (mm)  IGRA (IU/mL) | 26 (7)  0 [0-0]  1.14  [0.54-4.87] | 6 (9)  0 [0-0]  1.31  [0.52-4.87] | 7 (11)  0 [0-0]  1.93  [0.74-11] | 13 (5)  0 [0-0]  1.08  [0.49-2.60] | 0.39  0.67 | 17 (8)  0 [0-0]  1.20  [0.52-5.19] | 6 (8)  0 [0-5]  0.99  [0.87-1.93] | 3 (3)  0 [0-4]  2.04  [0.74-11] | 0.30  0.18 |
| **TST+/IGRA+**  n (%)  TST (mm)  IGRA (IU/mL) | 256 (67)  23 [19-27]  >10  [3.57 - >10] | 42 (62)  20.5 [19-25]  >10  [2.93 - >10] | 32 (52)  23.5 [17-27.5]  >10  [3.3 - >10] | 184 (72)  23 [20-27]  >10  [3.9 - >10] | 0.09  0.82 | 145 (67)  22 [18-26]  >10  [3.61 - >10] | 49 (62)  25 [20-27]  >10  [4.66 - >10] | 63 (72)  23 [19-27]  >10  [3.15 - >10] | 0.44  0.89 |

Values are median [interquartile range] or n (percent).

^*^ Group includes one patient with smear =negative and one smear =scanty.

AFB= Acid fast-bacilli; CFU= Colony forming units of *M. tuberculosis* in aerosols.

IGRA values are capped at 10. Those with values greater than 10 are reported as >10 for this analysis.
