# Supplementary material for: Cough Aerosol Cultures of Mycobacterium tuberculosis: Insights on TST / IGRA Discordance and Transmission Dynamics
Source: PLoS One. 2015 Sep 22;10(9):e0138358. doi: 10.1371/journal.pone.0138358 (PMC4578948; doi:10.1371/journal.pone.0138358)
Supplement: S3 Table — Legend: Values are median [interquartile range] or n (percent) * Group includes one patient with smear = negative and one smear = scanty AFB = Acid fast-bacilli; CFU = Colony forming units of M. tuberculosis in aerosols IGRA values are capped at 10 IU/mL. Those with values greater than 10, are given the value >10 for this analysis. “c” = conversion (from negative to positive); “nc” = non-conversion. (DOCX) [file pone.0138358.s003.docx]

**Table S3: Analysis of quantitative tuberculin skin test (TST) and interferon gamma release assay (IGRA) readouts at six weeks among contacts “at risk” for new *M. tuberculosis* infection (i.e. TST and/or IGRA negative at baseline) by exposure variable.**

| Contact category  Median [IQR] | Sputum AFB | | | Aerosol CFU | | |
| --- | --- | --- | --- | --- | --- | --- |
|  | 1+ | 2+ | 3+ | 0 | 1-9 | ≥10 |
| - n - | 13 | 17 | 41 | 37 | 19 | 15 |
| TSTnc / IGRAnc  n (%)  TST (mm)  IGRA (IU/mL) | 6 (46)  0 [0-0]  -0.04 [-0.05,-0.02] | 14 (82)  0 [0-0]  0.02 [-0.01, 0.08] | 20 (49)  0 [0-0]  0 [-0.06, 0.08] | 28 (76)  0 [0-0]  -0.01 [-0.05-0.05] | 10 (53)  0 [0-0]  0.03 [-0.05-0.09] | 2 (13)  0 [0-0]  -0.05 [-0.2-0.11] |
| TSTnc / IGRAc  n (%)  TST (mm)  IGRA (IU/mL) | 0 (0)  -  - | 1 (6)  0 [0-0]  0.54 | 4 (10)  0 [0-0]  0.79 [0.60-3.14] | 1 (3)  0 [0-0]  0.54 | 3 (16)  0 [0-0]  0.80 [0.77-5.47] | 1 (7)  0 [0-0]  0.43 |
| TSTc / IGRAnc  n (%)  TST (mm)  IGRA (IU/mL) | 3 (23)  18 [12-19]  0.09 [-0.03-0.14] | 0 (0)  -  - | 5 (12)  19 [12-21]  0.03 [-0.01-0.04] | 4 (11)  12 [12.0-16.5]  0.01 [-0.02-0.09] | 2 (11)  24.5 [19-30.0]  0.06 [0.04-0.07] | 2 (13)  18.5 [18-19]  0.03 [-0.03-0.09] |
| TSTc / IGRAc  n (%)  TST (mm)  IGRA (IU/mL) | 4 (31)  20 [20-22.5]  >10 [5.8 - >10] | 2 (12)  20 [20-20]  9.0 [7.1 - >10] | 12 (29)  22 [20.5-25.5]  >10 [>10 - >10] | 4 (11)  20 [17-20]  9.0 [5.4 - >10] | 4 (21)  23.5 [21.5-26]  >10 [8.3 - >10] | 10 (67)  22 [20-25]  >10 [>10 - >10] |

Values are median [interquartile range] or n (percent)

^*^ Group includes one patient with smear =negative and one smear =scanty

AFB= Acid fast-bacilli; CFU= Colony forming units of *M. tuberculosis* in aerosols

IGRA values are capped at 10 IU/mL. Those with values greater than 10, are given the value >10 for this analysis.

“c”=conversion (from negative to positive); “nc”=non-conversion.
